# Health care professionals’ experiences of caring for children with severe epidermolysis bullosa

**DOI:** 10.1177/20551029251391232

**Published:** 2025-11-05

**Authors:** Elisabeth Daae, Kristin Billaud Feragen, Terje Nærland, Charlotte von der Lippe

**Affiliations:** 1 Center for Rare Disorders, 155272 Oslo University Hospital HF, Oslo, Norway; 2 Institute of Clinical Medicine, 6305 University of Oslo, Norway; 3 KG Jebsen Center for Neurodevelopmental Disorders, Institute of Clinical Medicine, 6305 University of Oslo, Norway; 4 Norwegian Centre of Expertise for Neurodevelopmental Disorders and Hypersomnias, 155272 Oslo University Hospital, Oslo, Norway; 5 Department of Medical Genetics, Telemark Hospital Trust, Skien, Norway

**Keywords:** health care professionals, epidermolysis bullosa, genodermatosis, qualitative

## Abstract

Epidermolysis bullosa (EB) comprises a group of genetically and clinically heterogeneous disorders characterized by skin fragility and blistering. It is currently incurable, and care is complex because of the rarity of the disease. Epidermolysis bullosa has a major impact on the lives of people living with it and on their families. In this study, we aimed to explore health care professionals’ (HCPs) experiences of treating and following up on children with EB and their families. Nine HCPs from several health care disciplines participated in semi-structured interviews, which were analyzed through reflexive thematic analysis. Two main themes were identified: caring as an act of balance and facilitating collaboration. All participants experienced the following as challenging: (a) balancing between professional decisions and the provision of guidance to parents and (b) dealing with their own emotions. More systematic research is needed on the psychological impact of caring for children with EB and their families on HCPs.

## Introduction

Epidermolysis bullosa (EB) is a rare genodermatosis that results in fragile skin (Has et al., 2020a). It is a heterogeneous group of disorders that can be divided into four major groups: simplex, junctional, dystrophic, and Kindler (Has et al., 2020a). There is currently no cure for EB, and treatment includes extensive skin care, such as draining blisters, wound care, and pain alleviation (El Hachem et al., 2014).

Because the chronic fragility and blistering of the skin affect the physical, emotional, personal, and socioeconomic aspects of the lives of people with severe EB, multidisciplinary care teams are mandatory (Duipmans and Jonkman, 2010; Martin et al., 2019; Retrosi et al., 2022; Saad et al., 2024). This team should include nurses and doctors with different specialties, in addition to health care professionals (HCPs) educated in supporting patients and families regarding psychosocial issues (Retrosi et al., 2022). The aim of a multidisciplinary team is to administer proper diagnostic processes, holistic care, and treatment for people with EB; to provide therapeutic patient/parent education; and to help enhance quality of life by alleviating pain and supporting autonomy (Duipmans and Jonkman, 2010). Members of the multidisciplinary team and other providers experience frequent and long-term interactions with children and their families, and this will offer the possibility of providing patient and family-centered care. However, this can also present some challenges. As daily treatment and monitoring of symptoms are the parents’ responsibility, communication and a good relationship between parents and HCPs are important. This communication can be especially demanding emotionally for both sides when a genetic disorder is characterized by uncertainty and deterioration (Donohue et al., 2018), as in severe EB. Health care professionals caring for children with EB may have to deal with a variety of challenging feelings, such as overwhelmingness, fear, or sadness, because there is currently no cure for EB, and the alleviation of symptoms is only transitory (Chateau, Aldous, et al., 2023; Dures et al., 2010). This genetic condition presents a unique emotional burden due to the constant exposure to severe, visible suffering; inflicting pain through wound care; and the absence of curative treatment.

In the field of health research, the compassion and empathy expressed by HCPs can be mentally and economically costly (Cocker and Joss, 2016). Compassion fatigue can be an implication for HCPs and is described as a “healthcare practitioner’s diminished capacity to care as a consequence of repeated exposure to the suffering of patients, and from the knowledge of their patient’s traumatic experiences” (Huggard and Unit, 2013: 38). It is the result of giving care and compassion over a prolonged period to those who are suffering, often without experiencing the positive outcomes of seeing patients improve (Circenis and Millere, 2011). This situation has not yet been described or evaluated in HCPs caring for children with EB and their families, but the definition, which includes both repeated exposure to patients’ suffering and knowledge of how this affects the patients, indicates that HCPs might be at risk for developing compassion fatigue.

The interaction between parents raising children with EB and health care services needs to be based on trust and predictability. The parents of children with EB worry about HCPs’ limited knowledge about the condition, which may lead to potential delays in referrals to specialist centers (Dures et al., 2010). At the same time, parents’ lack of knowledge might result in more blistering and incorrect dressing of fragile and sensitive skin, which can lead to the deterioration of the child’s skin and/or health condition (Van Scheppingen et al., 2008; Yuen et al., 2012). Another barrier to seeking medical assistance for parents is that they do not feel that HCPs validate and acknowledge their opinions and knowledge (Kearney et al., 2020). Communicating well with parents is therefore a prerequisite for tailoring flexible care delivery models for children with EB and their families.

The immense medical and psychological needs of children with severe EB and their families challenge the current system in primary and specialist health care. Epidermolysis bullosa is a complex disorder requiring follow-up from many different disciplines, and guidelines have been developed to address the various health and social aspects of care, such as anemia management (Liy-Wong et al., 2023), cancer (Mellerio et al., 2016), foot care (Khan et al., 2020), hand surgery and therapy (Box et al., 2022), laboratory diagnosis (Has, Liu, et al., 2020), pregnancy and birth, (Greenblatt et al., 2022), palliative and end-of-life-care (Popenhagen et al., 2023), psychosocial issues (Martin et al., 2019), sexuality (King et al., 2021), wound care (Pope et al., 2012), and neonate care (Saad et al., 2024). These guidelines offer a useful framework for providing support and care to people with EB and their families.

Most studies on EB have focused on therapies and epidemiology, and very few have examined the impact of EB on HCPs (Chateau et al., 2023b). Health care professionals can experience the chronicity and the uncertainty of the disorder as stressful, and they may also witness the emotional impact on the parents and families of the person with EB, sharing a sense of helplessness and diminished hope (Dures et al., 2010). In addition to this, individuals with EB experience chronic and recurrent pain, which is difficult to alleviate (Goldschneider et al., 2014). Health care professionals caring for individuals with chronic pain are particularly at risk of developing burnout (Kroll et al., 2016; Rodrigues et al., 2018), potentially exposing HCPs caring for people with EB to risk. Because most children with EB now survive into adulthood and require lifelong follow-up, both HCPs and families need to receive the support they need in order to continue providing quality care.

The aim of this study is to gain an in-depth understanding of the subjective experiences of HCPs treating and following up on children with EB and their families, with the intention of identifying challenges and coping skills related to their care responsibilities and determining potential unmet needs.

### Organization and coordination of care for severe EB in Norway

The EB team, based at Oslo University Hospital, is a specialized service responsible for the care of individuals with the most severe subtypes of epidermolysis bullosa (EB) in Norway. The team consists primarily of dermatologists and nurses, and collaborates with other professionals—such as medical specialists, psychologists, social workers, and nursing consultants—when needed. Some individuals with the generalized form of EB simplex (EBS) are also followed by this team. Those with severe forms of EB are regularly called in for follow-up consultations and hospital admissions throughout their lives. The EB team provides comprehensive, long-term support to both the affected individuals and their families.

In the municipalities, the primary healthcare services are responsible for care. Healthcare professionals include home nursing services, general practitioners, physiotherapists, occupational therapists, and public health nurses. Before a newborn with EB is discharged from hospital, the EB team provides training to municipal HCPs to ensure they are prepared to support the family when they come home.

## Methods

### Study design and setting

We used a qualitative explorative approach with a semi-structured interview guide; this allowed for a systematic exploration of topics using predetermined open-ended questions, encouraging open-ended responses from the participants for in-depth information (DiCicco-Bloom and Crabtree, 2006).

The EB team cooperate closely with the Neonatal Intensive Care Unit (NICU), the Department of Gastroenterology, the Department of Child and Adolescent Mental Health, the Department of Plastic and Reconstructive Surgery, the Department of Clinical Nutrition, the Department of Pain Management and Research, and the Department of Physical Medicine and Rehabilitation. When a child with EB is discharged from the hospital, the primary health service is responsible for the follow-up of the child and the family. For severe forms of EB, local follow-up is done in cooperation with the EB team. The integration of care and communication between specialists and primary health care is paramount to ensure coordination and successful care transition. In this study, the term “multidisciplinary” is used to describe (a) collaboration within specialized or primary health care services, implying that physicians with different specialties interact and multiple HCPs with different professional backgrounds work together, and (b) collaboration across primary and specialized health care, also understood as collaboration across health care systems.

In Norway, municipalities are responsible for providing primary care to their populations, which is referred to as primary health care services in this study.

### Recruitment and sample

The Center for Rare Disorders (CRD), at Oslo University Hospital, is a national multidisciplinary resource center for several disorders, including EB. The CRD has a voluntary patient registry, which, at the time of this study, included 72 individuals with EB from all subtypes (25 individuals under 16 years old and 47 individuals aged 16 and above). The parents of all 25 individuals with EB under the age of 16 received a letter in February 2022 asking for their consent to invite HCPs from primary health care (those providing long-term follow-up) who will participate in the study. We received consent from three parents, who had children with severe EB, to invite eight HCPs. All HCPs were contacted, and three agreed to participate.

To recruit HCPs from specialist services, we sent an invitation to the EB team (*n* = 6) and the Department of Child and Adolescent Mental Health in Hospitals in 2020. The invitation to the Department of Child and Adolescent Mental Health in Hospitals was sent to one of the psychologists working with EB, and she was asked to share the invitation with colleagues potentially familiar with EB (*n* = 25). Two participants were recruited this way, and four from the EB team consented to participate. Furthermore, a total of nine HCPs, three from primary health care services and six from specialist health care services, agreed to join the research. They represented different professional backgrounds; they were nurses, doctors, social workers, and psychologists.

We asked for parental consent in primary health care services but not for everyone in specialist health care services, because primary HCPs had most likely followed up on only one child with EB, therefore making the data we gathered more sensitive than those obtained in specialist health care services. We also asked for consent from one of the HCPs in specialist care who had met only one family of a patient with EB prior to the interview.

### Data collection

The interview guide was based on existing literature and clinical experience. The interview guide was discussed with experienced CRD counselors with different specialties, who were also familiar with rare genodermatoses. Some of the questions from the interview guide used on HCPs from specialist services were slightly changed to tailor the questions and better match the area of responsibility for follow-up of primary health care.

The interviews were conducted by the first author, ED, from April 2020 to September 2022. All interviews with the participants from specialist health care services were conducted face to face, whereas the interviews from primary health care services were conducted over the phone because of geographic distance. On average, the interviews lasted for 45 minutes (range: 31–57 minutes) and were audio-recorded and transcribed verbatim.

### Analysis

We adopted reflexive thematic analysis using the six stages of Braun and Clarke (2019). In the first step, we read all interview transcripts to familiarize ourselves and engage with the data and to identify appropriate information that may be relevant to the research question. The interviews were coded manually on a semantic level (inductive, data-driven codes using the participants’ own words, identified through the explicit meanings of the data). The initial codes were broadly defined to bring together a group of data excerpts that could be related. We then organized the data extracts into different codes, and an overview of the codes was made for each interview. All authors discussed the main tendencies within the dataset. The analysis involved making sense of the relationships between the different codes by moving back and forth between the excerpts. Finally, two themes were identified.

### Reflexivity

Reflexivity was an important part of the entire research process. ED, KBF, and CvdL are all familiar with EB. ED has been involved in work on EB at the CRD since 2009, such as lectures during educational conferences for families living with EB, international conferences on EB, meetings with the national patient association, and close collaboration with the national EB team for several years. CvdL has more than 20 years of experience as a clinical geneticist, including genetic counseling to families with children affected by EB, and extensive research experience. KBF is a clinical psychologist and researcher with the psychosocial impact of living with a visible difference as her area of expertise, which has provided some familiarity with EB.

It is equally important to note that our knowledge and experience of EB helped us identify areas that merited further probing during the interviews. They were particularly useful during the analysis, as they helped us understand and recognize the experiences described by the HCPs. However, this could have also potentially caused our interpretations of the interviews to be biased. To enhance credibility and counteract group thinking and researcher bias, the first and last authors comprised the primary analytic team. The third author, TN, is an experienced researcher and psychologist who is not familiar with EB but is familiar with other rare disorders, so he served as a discussant. We were able to benefit from the different positions we represented, and we encouraged reflections on our different backgrounds and experiences.

### Ethical considerations

Ethical approval for the study was obtained from the Ethics Committee (South East Norway, reference number 2019/567) and was accepted by Oslo University Hospital’s Data Protection Office (reference number 19/08354). This study was conducted in accordance with the Helsinki Declaration. All parents provided written consent to invite HCPs who had treated their children in the primary health care services to participate, and they provided the names, professions, and telephone numbers of the HCPs they consented to invite. HCPs also gave written consent. Prior to each interview, the first author explained the study’s purpose and regulations regarding confidentiality and specified the participants’ rights to withdraw their consent at any point until submission.

## Results

Two main themes were identified: caring as an act of balance and facilitating collaboration. Table 1 shows the main themes and subthemes.

**Table 1.** Main themes and subthemes.

Main themes	Subthemes
Caring as an act of balance	Communication
	Building parental confidence
	Handling personal emotions
Facilitating collaboration	Shared responsibility
	Psychosocial support for healthcare professionals

Representative quotes were selected to generalize the descriptions of the participants’ experiences. The participants were referred to as *primary HCP* or *specialist HCP* to preserve their anonymity.

### Caring as an act of balance

The participants highlighted that taking good care of children with EB also implied taking care of these children’s parents. There were several aspects of caring for the family that needed careful monitoring, such as how much practical and emotional responsibility the child or the parents could handle and the kind of information they needed at different times.

#### Communication

The HCPs reported that several of the parents had mother tongues other than Norwegian and that their fluency in Norwegian varied. The parents were usually offered to use a professional interpreter to ensure that they and the HCPs understood each other during conversations/consultations. Some of the parents declined the proposal for different reasons, such as believing that their Norwegian skills were fluent enough or that enough people were already involved. A consequence of having consultations without an interpreter is that a parent with good Norwegian language proficiency could possibly take the lead in the discussion. Other parents could then experience difficulties speaking up, and it may be challenging for the HCPs to identify misunderstandings or disagreements between the parents. The HCPs also chose not to use an interpreter in some situations. Because there were considerable differences in how EB affected the children’s skin, the HCPs could have felt insecure during skin care and, owing to this, did not bring a professional interpreter into that situation because they needed to stay fully focused on the procedures. Health care professionals chose to involve a professional interpreter in other situations, such as diagnostic conversations. However, if an interpreter was used, some of the HCPs reflected on the need for debriefing the interpreters because of how frightening the disease could sometimes appear.

“To an interpreter, to translate information about a child they’ve never met, about a disease they know nothing about, which maybe Googled a couple of minutes upon meeting the family, being exposed to all these horrific images—it’s pretty harsh”. (Specialist HCP)

All the interviewees from specialist health care services reported that they experienced the immediate time after birth as particularly disturbing to the parents, and this initiated some ethical dilemmas for the EB team. The exact diagnosis could remain unclear right after birth because the genetic cause was pending, and conversations with the parents at that point were described as “difficult conversations.” This uncertainty was a challenge for both the HCPs and the parents. As some of the subgroups of EB are lethal and difficult to separate from less severe subtypes in the immediate period after birth, the HCPs were concerned about how to communicate with the parents and be realistic while at the same time allowing the parents to remain hopeful that their children’s EB is of a less severe type.

“It’s an act of balance, to be clear and concise, but at the same time [we strive to] help the parents not to fall apart.” (Specialist HCP)

Another dilemma experienced by members of the EB team was the amount of information that should be shared with the parents immediately after the child’s birth. They experienced that some of the parents wanted much information, whereas others did not want to take in more information than absolutely necessary. Some parents struggled to absorb information because of high levels of stress. The HCPs preferred that the parents consider them as primary sources of information, rather than resorting to the internet. The participants reported that most of the parents had found pictures of severely affected infants by searching on the internet on their own, which led to increased stress levels and anxiety in the parents.

“We must give correct information but not too much. It might easily become too much.” (Specialist HCP)

The interviewees from specialist health care services observed that the parents of children with severe types of EB were more affected psychologically than the parents of children with milder EB types. The severity of the disease affected the communication process. The HCPs described how they tried to be more tactful in their choice of words create tension when conversing with the parents of severely affected children to avoid burdening and scaring them further.

The HCPs in primary and specialist health care services reported that the parents reacted differently to their children’s diagnoses, and they were not necessarily ready at the same time, to learn about EB. This could potentially create tension in the relationship between parents. At times, HCPs considered it wise to communicate with the parents separately. Witnessing the tension between parents and balancing the need for information versus the need to get used to the situation at their own pace was a challenge:

“The mother took a step back. It became too much for her, but the father took a step forward and wanted to know everything (…) he sought guidance and information. He became alone; it was a lot to deal with for him. I’m not sure how the mother experienced this, if it became an extra burden for her that the father was the stable caretaker, as she had more than enough dealing with herself and her emotions.” (Specialist HCP)

The participants from the EB team stated that they experienced good communication with children below the age of 10 years. However, the children slowly became more reluctant to disclose their thoughts and feelings when they reached the age of 10–12:

“I noticed that the older the children get, some of the children I used to communicate well with, I don’t connect as well with anymore. They wouldn’t, couldn’t take it anymore.” (Specialist HCP)

When this happened, the HCPs depended on developing/having a good dialogue with the parents to stay informed about the children’s physical and mental health. A few HCPs assumed that the children were worried about dying from EB at a young age, but they did not raise this issue directly with the children.

“They probably realize that they won’t get very old, and they observe other children at their age running around, playing football, and doing stuff children do, but they can’t join, and maybe they find that difficult.” (Specialist HCP)

There were also some issues that were not voiced explicitly by the parents according to the HCPs, but these issues concerned the HCPs themselves. One of these was communication about the disorder within the family—how family members talked about it and what the child knew about EB and his/her expected progress/deterioration. As skin care is the main treatment for EB, which was mainly performed by the parents, the HCPs needed to know how this treatment was being done at home. When the interviewees described how the parents talked with the children about EB and how EB affected the patients and their families, the HCPs used words such as “think,” “assume,” and “under the impression.” This could indicate that the HCPs did not raise these topics during consultations with the families and the children.

“I experience that the families talk about EB, but maybe more so when the child isn’t present, but I don’t know how they communicate.” (Specialist HCP)

The HCPs in primary health care services reported the same. They found it difficult to address some issues they felt that the parents were reluctant to discuss. Examples of this were the parents’ mental health and how this could affect interaction with the child, consanguine marriages, and raising a child with a severe genetic skin disorder. This was evident when the parents did not answer questions or ask questions pertaining to these issues.

“There aren’t many answers, or there’s a feeling that they won’t share so much (…) it wasn’t natural in a way to raise questions that are taboo or induce shame/guilt.” (Primary HCP)

A few from specialist health care services stated that there were also some topics that were not addressed in the consultations, unless the parents raised them. Examples of this were difficulties related to hanging out with friends and other psychosocial consequences of EB. This did not mean that the HCPs did not consider such concerns, but they thought that it was time consuming to bring these up because they knew this was important to the families. Another topic was birth control. The HCPs thought that this should be important for the parents because they just had a child with a genodermatosis, making heritance, genetic testing, and birth control highly relevant issues to address. However, the HCPs were not always sure whether the parents would like to discuss this issue.

“It was probably a topic to them (the parents) mentally, but I’m not sure they’d like to talk about it.” (Specialist HCP)

A few of the HCPs from primary health care services said that they also used intuition and their gut feelings in their consultations with families parenting children with EB.

“Maybe we use our senses more than we assume, sometimes, just to understand when it’s ok to move on or not, what we may ask them, just to find our way in the communication.” (Primary HCP)

#### Building parental confidence

The HCPs communicated that having a child with EB demanded that the parents create a new *parental coping toolbox*. This also applied to the HCPs, as they did not have all the answers and had to learn by trial and error together with the parents. Attempting to connect with a child with EB as a parent was also one of the first dilemmas that the HCPs observed. Some HCPs had experienced that the parents found it emotionally stressful to look at their children’s skin, and the HCPs then wondered whether this affected the parents’ emotional bonds with their children. They described how some parents struggled with spending time with the infant immediately after birth, and they worried that the parents’ fears and emotions could get in the way of caring for the child. Some HCPs were concerned about when they should gently push the parents to take part in skin care and when to reassure them and let them keep their distance from the painful parts of the infant’s care. Health care professionals from the Department of Child and Adolescent Psychiatry tried helping parents develop new coping skills by understanding and communicating with the children, using their (the parents’) voices, and observing the children’s temperament and how the infant signaled hunger and other needs.

“In the beginning, we had to work hard to make the mother feel safe enough to spend a little time with the child, first, by watching the baby for short periods of time, within the limits of what she could endure, and then gradually make her feel safer.” (Specialist HCP)

When the parents and the children with EB were discharged from the hospital, the EB team and HCPs from other departments helped prepare them for their new everyday lives. However, the HCPs in specialist health care talked about how difficult they felt it was to know which parenting resources would normally be available to parents, as some of these parents were in the middle of a crisis and therefore had less access to their usual coping strategies.

“In one of the families I met, the diagnosis hit them like a bombshell and destabilized the mother completely, for a long time, because it came as an absolute shock to her. She showed symptoms of being in a major crisis. She completely lost herself and her dreams for the future”. (Specialist HCP).

The EB team tried to plan home health care for the long run, and not just for the immediate future. They stressed that, although the families went through a crisis, they should have not only their own space to create as much normality as possible to establish a family life but also as much support as necessary to be able to cope with the demands of EB. What was perceived as helpful to the families could vary, and the balance between having too many HCPs and just enough HCPs in and out of their own homes was not always easy to decide.

When the parents finally felt safe taking responsibility for their children’s skin care, the HCPs reported that the parents had difficulties accepting help from others. Interviewees from primary health care services had also experienced that some toddlers refused skin care from people other than their parents.

“You can’t force your help on a child who really opposes, so then the parents end up performing skin care themselves.” (Primary HCP)

Health care nurses in primary health care experienced that families raising children with EB benefited from home visits. In this way, the parents needed to plan less and move less between their homes and the community health center. The HCPs also communicated that it became a natural and important distinction, meaning that home visits indicated more focus on the child and the parents, and less physical examinations had to be performed compared to visits in the community health center.

#### Handling personal emotions

Health care professionals from the specialist health care services said that it was emotionally draining to spend time with the infant right after birth and with the infant’s family, as dealing with parents who could be devastated and have severely ill children was difficult.

“I think it’s a tough situation to walk in to… opening up to infants emotionally, relating to them is easy, but it’s costly. It costs to witness a parent pushing away small children; it costs to see totally devastated parents. It does affect me.” (Specialist HCP)

All interviewees agreed that it was emotionally demanding to work with children and families with EB. At the same time, they considered it unprofessional to reveal their true feelings to the parents, so they had to try to keep their feelings hidden. Other HCPs experienced that it was emotional to work with children with EB but that they succeeded in keeping their feelings under control.

“You’re a professional, but at the same time there are personal feelings as well, and you do sympathize with the family. It’s horrible to watch what the child must endure… but it’s not like the feelings take control.” (Primary HCP)

The aspect of disease chronicity was difficult for all interviewees to handle. A few in the specialist health care services reported that it was emotionally tough for them to learn that a child had a reduced life expectancy. They found it hard to stop thinking about these children even when they came home from work. It added to the burden when they identified themselves as parents, and not just HCPs. They had cared for children with severe EB and knew that some of the children would potentially not survive past the first year of life, while other children faced the risk of experiencing long-term complications and skin cancer.

“It crosses my mind frequently that when some of the young adults get worse, I know that I’ll follow them to the end of this life, and that’s going to be difficult when you know them so well.” (Specialist HCP)

All the interviewees from specialist health care services also stated that the children’s anxiety levels increased as they grew older, and this coincided with the children’s reluctance to disclose their feelings and thoughts. The HCPs observed that it could become more and more difficult to take blood samples and that older children tended to be more afraid of needles. Some children needed general anesthesia for simple procedures, such as drawing blood samples. This gave HCPs a feeling of failing to help the children. The EB team experienced this as emotionally demanding—trying out different solutions, not being able to evaluate directly how these worked out with the child, and learning whether the HCPs’ attempts at interaction and communication were rejected.

### Facilitating collaboration

The EB team, the Department of Child and Adolescent Mental Health, and primary health care services all described cooperation as an important part of their follow-up with children with EB and their families. They said that they could not take care of every need of the families and that they depended on other HCPs to provide holistic care. This theme describes what HCPs experienced as helpful in facilitating multidisciplinary collaboration.

#### Shared responsibility

Prior to a family’s discharge from the hospital, the doctors and nurses from the EB team invited the parents and the HCPs from primary health care services so that they could learn skin care. The latter observed the EB nurses in interaction with the children and practiced some necessary skills, such as puncturing and draining blisters. These preparations for learning skin care and meeting the child and his/her family were described as positive for all parties.

“In the meeting I attended in the hospital, we were given a thorough explanation of EB, which was very good, and I was given an opportunity to call specialist health care services later when necessary. It felt very reassuring.” (Primary HCP)

The EB team stressed the importance of finding dedicated HCPs in other departments in specialist health care services, such as the NICU, to facilitate collaboration. In this way, dedicated HCPs could gain knowledge about the disorder and the individuals with EB, and it was easier to ensure continuity. If a child with EB was born during holidays in which continuity was difficult to pursue, the EB team tried to maintain continuity by working longer hours so that the families would meet some familiar faces and to counteract the turnover of HCPs in other departments.

The HCPs working in the EB team reported that collaboration between the NICU and the Department of Child and Adolescent Psychiatry was highly satisfactory, and that they were also pleased with how the workload was divided among them. They emphasized that referrals to local Departments of Child and Adolescent Psychiatry were more easily accepted when the Department of Child and Adolescent Psychiatry at Oslo University Hospital wrote the referrals. They already knew the families, and they could use their own terminology to describe why they needed further follow-up at home. Therefore, the EB team could spend time on skin-related issues instead.

The HCPs also appreciated collaboration between the EB team, the NICU, and the Department of Child and Adolescent Psychiatry. Knowing other HCPs and being aware of their expertise facilitated cooperation across units. Collaboration was needed when HCPs in one of the units, for instance, required expertise from another unit, such as when expertise about EB had to be combined with the general counseling of the parents.

All interviewees agreed that it was essential that the HCPs cooperated closely to ensure that everybody communicated the same messages about EB to the families.

“I think that in cases like these (families with EB), it must be a clear advantage to create multidisciplinary teams at the birth of the child so that it’s possible that we can share all experiences, see the whole family, the totality of their experiences, all nuances of that.” (Specialist HCP)

The HCPs in primary health care wanted closer collaboration with other HCPs in primary health care to ensure comprehensive care for the child with EB and his/her family. Health care nurses experienced sparse contact with home nursing care. Home nurses contacted the general practitioner or specialist health services if they had any questions pertaining to EB. For HCPs in primary health care, contact with specialist health care was especially important. They stressed that it could not be expected that they had specific knowledge about EB before they met a child diagnosed with it, because it is a rare disease.

Health care professionals working in the EB team also stressed the importance of staying up to date with research on the disease and its treatment. They appreciated the clinical practical guidelines developed for EB.

“It’s much easier to take care of the child (when you have guidelines) and argue for giving evidence-based treatment.” (Specialist HCP)

#### Psychosocial support for HCPs

The EB team, the Department of Child and Adolescent Psychiatry, and primary HCPs acknowledged their own need for psychosocial support, and they found support in one another. The nurses never performed skin care alone; they were always in pairs. However, nobody had systematic psychosocial support; they had to ask for support themselves. All interviewees recognized the need for support.

“EB is a ‘heavy’ patient group to work with. It’s difficult to work with them all the time, with really sick children. I also see other skin patients because I need to, I have to.” (Specialist HCP)

This quote illustrates the emotional burden of working with EB patients. Alternating with other patient groups serves as a form of emotional relief from the intensity of caring for severely affected children. The HCPs in both primary and specialist health care mentioned flexible solutions as important. Debriefing was required in more demanding situations, such as the death or deterioration of a patient. Therefore, the HCPs needed more support in these extraordinary situations than in everyday care. They wanted to have the possibility of asking for help when they felt the need for it, instead of regular debriefing with professionals other than their closest colleagues. Some HCPs from the EB team highlighted conversations with a priest as helpful. Health care professionals from primary health care described asking colleagues for debriefing or support, depending on the resources available. In offices characterized by a scarcity of resources, particularly in primary care, it was more difficult to ask for support and help and to express the will to help when asked. The rarity of EB was mentioned as a complicating factor because it was time consuming to explain the diagnosis and its needs to colleagues who were not familiar with it.

“We are so few, and the demands are so many. So yes, we can discuss cases, but when you’re not involved with that child, it’s difficult to map out and understand the needs.” (Primary HCP)

Working with colleagues from different professional backgrounds was also mentioned as helpful by the HCPs in specialist care. The nurses who were frontline providers of care were closest to the families in every way, and they mentioned that the more theoretical approach the doctors adopted when meeting with these families could serve as a tool to help them (the nurses) create some emotional distance. Getting to know one another’s strengths and weaknesses as humans was also described to be important in determining who was best at solving which issues.

“She’s really good at communicating with the parents and children, but if there’s discussion about the administrative part of our work, like what we should prioritize to follow our budget, I’m happy to solve those issues.” (HCP specialist care)

## Discussion

The aim of our study was to explore HCPs’ experiences of working with children with severe EB and their families. Health care professionals described situations in which they had to balance how they provided care by monitoring the amount of information they gave to the parents, handling the emotions that the child with EB and his/her family evoked in them, and adjusting to the perceived needs of the child and the family. The parents’ proficiency in Norwegian, the severity of the disease, how the parents reacted to their children’s EB diagnosis, and the child’s age and experiences of living with EB could affect the communication process. Health care professionals also wondered whether the shock that came with the child’s EB diagnosis in some families could affect the parents’ coping skills, and this complicated the HCPs’ assessment of which resources the parents had access to within themselves. The HCPs found it emotionally challenging to work with devastated parents and chronically ill children in pain. Building multidisciplinary collaboration between specialist and primary health care services was perceived as central, and psychosocial support from other HCPs, such as their colleagues, was considered important to be able to cope with the emotional impact of working with children with EB.

The HCPs from specialist health care services in our study met the parents in an extremely vulnerable situation—while their children were admitted to the NICU and while probably experiencing loss of parental control (Obeidat et al., 2009) and facing the unexpected possibility of a genetic disease in their children. Therefore, there were possibilities that the parents may be in shock. Learning to care for a child with EB with symptoms present from birth can be challenging. Parents need to acquire new, unfamiliar health care-related skills and incorporate them into their parenting toolkit. Health care professionals should help parents gain parental confidence by highlighting positive aspects of parenting and providing them with sufficient information and the necessary skills to address and take care of the children’s medical needs. While the parents and their children are getting to know one another, a medical process is taking place in parallel. Disclosing the disease-causing gene variants in EB is essential for early prognostication of the disease course, genetic counseling, and future precision medicine approaches (Has et al., 2020b). An exact diagnosis could help redirect care toward palliation, when necessary, for individuals with severe EB (Yang et al., 2014). The time before a clear and exact diagnosis was made was difficult for both HCPs in specialist health care and the parents because of the possibility of a fatal outcome. All HCPs in specialist health care stated that the parents were understandingly anxious while they waited for the genetic test results. Conversations disclosing genetic test results will often include a discussion of the expected prognosis for the child and how the functional impact of EB can be expected to affect it. Prognostic communication can represent a challenge to HCPs, especially when the prognosis is poor or less certain (Mueller et al., 2023), as evident in our study. They strived to find a balance between giving enough and too much information, and they were highly concerned about the words to use in order not to frighten the parents.

The HCPs should be aware that parents vary in how much information they want and are able to process about the diagnosis initially (Haward et al., 2020). Before entering these conversations, HCPs should carefully consider whether to align with parents’ preferences for knowledge or reveal information that they think the parents need to know no matter what; they should prepare for anything ranging from revealing little information to discussing typical disease trajectory honestly and in detail (Lemmon et al., 2023). Cognitive and emotional overload in the parents may occur because prognostic communication could affect their dreams, hopes and fears (Mueller, 2023). Although uncertainty could be distressing to parents, it may, in some cases, serve as a source of hope. Making the parents remain hopeful was important to the HCPs in our study. This finding is similar to that in the case of children with rare neurologic disorders in another study (Bye et al., 2022). Some common traits between rare pediatric neurologic disorders and severe EB subgroups are the unpredictability of symptoms, demanding home care, deterioration, and pain, which significantly affect children with EB and their families. Health care professionals can help with relief, but they cannot cure the disease. They can help with reducing the child’s stress, and they should also pay attention to the parents’ psychological needs. Some HCPs may feel insecure regarding how uncertainty should be handled professionally (Simpkin and Schwartzstein, 2016), therefore making the clinical management of hope a challenge. In the case of EB, the results of genetic testing can have significant implications for the child and his/her family, and this is a well-known fact for the EB team.

Epidermolysis bullosa is a chronic disease, and communication between the child, parents and HCPs is vital to delivering high-quality care. Communicating openly about progressive genetic disorders with children can be difficult because of the emotional toll on all family members (Sætrang et al., 2019). The progressive nature of severe EB may need to be disclosed gradually, and HCPs could act as facilitators of this communication between children and their parents and siblings. In our study, the HCPs found it more challenging to talk with the children as they became older. They experienced that children became reluctant to disclose their thoughts and feelings as they matured, and the HCPs described that the timing of this reluctance often concurred with the child’s realization that they suffer from a severe, chronic skin disease. At this age, around 10 to 12 years, the child should prepare for transition and self-care, and the HCPs described it as a paradox that the parents had to do the talking for the children. This is the age at which the child becomes aware of his/her illness, which could be traumatic insight for him/her (Retrosi et al., 2022). Genetics may often be a focus in initial diagnostic conversations, but researchers have paid less attention to how prognostic communication is provided by HCPs or within families as the child gets older (Mueller et al., 2023). A study of adolescents and young adults with EB indicated that some children with the severe EB forms experienced that their parents prevented them from participating in some activities because of the risk of being injured (Sangha et al., 2021). It was, however, not described how the families communicated about these issues. The HCPs in our study did not ask parents directly about how they communicated about EB in their families. In Norway, families are offered psychosocial support from professionals such as psychologists, social workers, or mental health-trained nurses, but access depends on the family’s informed consent. This may delay support if families are hesitant to engage or unaware of its relevance. Clinicians seem to leave the responsibility to the parents, who, in turn, might not feel equipped or supported. Part of this problem could possibly be explained by a gap in communication flow between primary and specialist health care services, leading to a fragmented distribution of tasks. Role clarification and outlining clear responsibilities in each case could improve the coordination of health care and communication. This line of responsibility should not be static but should be discussed regularly with every parent raising a child with EB so that they can learn the prognostic implications of EB and facilitate the development of autonomy.

The HCPs in our study had mixed experiences with using an interpreter. Some members of the EB team found it challenging to use an interpreter during skin care immediately after birth. They experienced it as a sensitive, chaotic setting because of the many professionals involved, in which the EB team did not feel comfortable before getting to know the child’s skin and symptoms better. One could question whether the lack of an interpreter left the parents more apprehensive than necessary, as they possibly did not yet fully understand the diagnosis, the symptoms, or the treatment. However, health care professionals used an interpreter in meetings with parents outside of the NICU. Using an interpreter in only shorter periods in just some of the conversations with the parents could possibly influence HCPs’ ability to build a close connection with the parents because the parents and the HCPs are deprived of caring conversations and small talk between formal meetings. At the same time, it could be difficult for the HCPs to attend to parents’ emotional and/or psychological needs when they lack a common language. Cultural differences can hamper and affect neonatal decision making (Van McCrary et al., 2014), possibly resulting in miscommunication, mixed messages, denial, or family reliance on information from the internet.

All interviewees in our study found it emotionally challenging to work with children with EB and their families. The act of suppressing emotions in the workplace in order to follow the organization’s rules is called emotional labor (Grandey et al., 2012). Nurses in the EB team are frontline providers of care, and the family’s and the child’s needs are intense. For nurses in both primary and specialist health care, caring for patients involves emotions, but the expression of emotions is also necessary in order to show empathy (Karimi et al., 2014). Nurses can experience challenges when they become too close to or too distant from the families (Stayt, 2009). In our study, the nurses in the EB team acknowledged the emotional demands of caring for children with EB and their families. They expressed that at times, creating the necessary emotional space between them and the families so that they can also take care of themselves can be difficult; by contrast, the HCPs in primary health care services felt that they did not get close enough to the families emotionally to be able to identify unmet psychosocial needs.

Families raising children with severe subgroups of EB need to relate to many different providers, and they may have to choose to whom they confide. The HCPs in our study expressed distress and discomfort but did not seem to have avenues where they could find emotional support. Health care professionals in contact with children with EB and their families over a long period of time are exposed to prolonged provision of patient care, with substantial exposure to “vicarious trauma” (Cavanagh et al., 2019), defined as witnessing the trauma of others, particularly of the children and their parents in our study. This could represent a risk factor for compassion fatigue. Health care professionals working with children affected by severe EB witness that their care helps lead to a potentially longer life expectancy, but at the same time, this also implies a longer/prolonged period of care. Whether a prolonged period of care means a greater emotional impact on HCPs is not explored in this study, but it could be assumed that this also implies an even closer relationship with the child and his/her family, making emotional labor even more demanding. Further research is needed to clarify whether HCPs experience compassion fatigue and to possibly develop interventions to alleviate it.

### Strengths and limitations

This study involved few participants, particularly from primary health services, but given the low number of professionals working with children and families with EB and the choice of a qualitative methodology, we can say that the sample size was acceptable. We also needed consent from the parents to invite HCPs from primary health care services, which restricted the HCPs whom we could contact. A broad sample of HCPs treating all subgroups of EB would have been valuable. Nevertheless, this study involved staff from different clinical settings, including different hospital departments, and from a larger geographical area, which may have provided important and valuable insights into national and regional care. Another limitation is that the HCPs in our study primarily treated children with severe EB, which meant that HCPs treating milder forms of EB were not given a voice. The fact that interpreters were not necessarily included when communicating with the parents may have also led to misunderstandings in the clinical setting. This could have affected the HCPs’ understanding of how the parents coped with their situations, so the HCPs just made their own personal interpretations of what was going on in a family when they were asked about it in the interviews. Overall, our study addresses the need for further research on HCPs caring for children with EB and their families.

## Conclusions

The aim of our study was to explore HCPs’ care responsibility experiences when following up on children with EB and their families. The HCPs described many situations in which they had to balance the care they provided to the children and their families, such as how to build up parental confidence, how to communicate with the parents, how to communicate with the growing child, and how to follow up with the families, which affected the HCPs emotionally. Caring for a child with EB professionally demanded multidisciplinary collaboration within and across specialist and primary health care to address families’ needs comprehensively. A multidisciplinary approach should also be pursued to ensure cooperation between primary and specialist health care, as well as within hospital settings.
